# Regulation gene expression of miR200c and ZEB1 positively enhances effect of tumor vaccine B16F10/GPI-IL-21 on inhibition of melanoma growth and metastasis

**DOI:** 10.1186/1479-5876-12-68

**Published:** 2014-03-14

**Authors:** Xiaoying Wang, Xiangfeng He, Fengshu Zhao, Jing Wang, Hongyi Zhang, Fangfang Shi, Yunxia Zhang, Kai Cai, Jun Dou

**Affiliations:** 1 Department of Pathogenic Biology and Immunology of Medical School, Southeast University, Nanjing 210009, China; 2 Department of Medical Oncology, Affiliated Tumor Hospital of Nantong University, Nantong 226361, China; 3 Department of Gynecology & Obstetrics, Zhongda Hospital, Medical School, Southeast University, Nanjing 210009, China; 4 Department of Oncology, Zhongda Hospital, Southeast University, Nanjing 210009, China

**Keywords:** Melanoma, Tumor vaccine, Interleukin −21, miR200c, Zinc-finger E-box binding homeobox 1

## Abstract

**Background:**

Genetically modified cells have been shown to be one of the most effective tumor vaccine strategies. However, in many cases, such as in melanoma, induction of a potent immune responses against the disease still remains a major challenge. Thus, novel strategies to reinforce tumor vaccine efficacy are needed. Using microRNA (miR) and Zinc-finger E-box binding homeobox (ZEB) have received much attention for potentially regulating tumor progression. To elicit a potent antitumor efficacy against melanoma, we used tumor vaccine in combination with miR200c overexpression or ZEB1 knockdown to assess the efficacy of treatment of murine melanoma.

**Methods:**

B16F10 cell vaccine expressing interleukin 21 (IL-21) in the glycosylpho- sphatidylinositol (GPI)-anchored form (B16F10/GPI-IL-21) were developed. The vaccine was immunized into mice challenged by B16F10 cells or B16F10 cells stably transduced with lentiviral-miR200c (B16F10/miR200c) or transfected with the ZEB1-shRNA recombinant (B16F10/shZEB1) or the B16F10/GPI-IL-21 vaccine. The immune responses, tumorigenicity and lung metastasis in mice were evaluated, respectively.

**Results:**

The vaccination with B16F10/GPI-IL-21 markedly increased the serum cytokine levels of IFN-γ, TNF-α, IL-4 and decreased TGF-β level as well as augmented the cytotoxicity of splenocytes in immunized mice compared with control mice. In addition, the tumor vaccine B16F10/GPI-IL-21 significantly inhibited the tumor growth and reduced counts of lung metastases in mice challenged by B16F10/GPI-IL-21, B16F10/shZEB1 and B16F10/miR200c respectively compared with the control mice challenged by B16F10 cells. The efficacy mechanisms may involve in reinforcing immune responses, increasing expression of miR200c, E-cadherin and SMAD-7 and decreasing expression of TGF-β, ZEB1, Vimentin and N-cadherin in tumor tissues from the immunized mice.

**Conclusions:**

These results indicate that the tumor vaccine B16F10/GPI-IL-21 in combination with miR200c overexpression or ZEB1 knockdown effectively inhibited melanoma growth and metastasis a murine model. Such a strategy may, therefore, be used for the clinical trials.

## Introduction

Melanoma is malignant and is one of the deadliest forms of skin cancer and its incidence is expected to rise over the next two decades. Despite significant progress in melanoma research having been made over the years, there are no effective therapies for advanced melanoma at present [1,2]. A growing body of literature shows that the immunotherapeutic approaches to use the immune system against cancer have focused on therapeutic vaccines that intended to specifically initiate or amplify a host response against evolving tumor cells [3-5].

Glycosylphosphatidylinositol (GPI) is a posttranslational added lipid anchor and GPI-anchored membrane cytokines have been shown to play an important role in host immune responses against tumor cells [6]. Interleukin (IL)-21 is a T cell-derived cytokine that is involved in T and NK cell activation in the tumor vaccine approaches. Studies have showed that IL-21 is widely applied to significantly augment antitumor immunity in multiple murine tumor models and clinical trials [7-9], and to generate considerable research interest in understanding its mode of action. Our previous study showed that administering whole tumor cell vaccine expressing IL-21 in the GPI-anchored form (B16F10/GPI-IL-21) induced protective anti-melanoma immunity in a B16F10 cell transplantable mouse model. However, an anti-melanoma efficacy failed to completely induce objective tumor shrinkage because the measurable and metastatic tumors were developed in some B16F10 melanoma-bearing mice, highlighting the need for using combination and alternative strategies [10,11]. More recent approaches have centered on a series of molecules known as potentially regulating tumor progression such as microRNA-200c (miR200c) [12] and zinc finger E-box binding homeobox 1 (ZEB1) [13], which have provided proofs that overexpression of miR200c or knockdown of ZEB1 could repress tumor ‘epithelial to mesenchymal transition’ (EMT) program that activates cellular mobility, subsequent tumor metastasis [14-16].

Recent researches have demonstrated that ZEB1 has an important regulatory role on the malignant tumor progression through regulating cell cycle, apoptosis, senescence, invasion, metastasis and mesenchymal angiogenesis [12,17]. Our previous studies indicated that the disturbance of EMT-miR-200c-ZEB1 feedback loop promoted the melanoma proliferation and invasive metastasis [16], and that the miR-200c overexpression in CD44^+^CD133^+^B16F10 cells markedly inhibited the cell proliferation and invasion ability *in vitro* as well as tumorigenicity *in vivo*[18]. It is known that the rapid progression of primary melanomas to locally invasive and metastatic other sites is a major obstacle for therapeutic interventions of melanoma patients. On the basis of these findings, we speculate that the EMT-inducer ZEB1 may support tumor metastasis in B16F10 melanoma-bearing mouse model by promoting tumor cell mobility and dissemination, and that antitumor immune responses induced by tumor vaccine B16F10/GPI-IL-21 might result in tumor cell apoptosis and inhibit tumor growth but do not block the EMT of B16F10 cells or metastasis in mice. In this regard, we used the novel strategy that the tumor vaccine B16F10/GPI-IL-21 was combined with overexpression of miR200c or knockdown of ZEB1 to evaluate the synergitic efficacy of treatment of murine melanoma, and to attempt to investigate the molecular mechanisms of melanoma metastasis in the present study.

Here, we show that the tumor vaccine B16F10/GPI-IL-21 in combination with potentially synergistic active therapies, such as either miR200c overexpression or ZEB1 knockdown, significantly represses tumor growth, blocks melanoma EMT program and inhibits tumor metastasis in a murine melanoma model.

## Materials and methods

### Animals

C57BL/6 mice between 5–6 weeks of age were obtained from the Yang Zhou University of China (license number: SCXK, Jiangsu province of China, 2007–0001). All mice were housed under the specified pathogen-free condition and the experiments were performed in compliance with the guidelines of the Animal Research Ethics Board of Southeast University.

### Cells

B16F10 murine melanoma cell line is syngeneic in C57BL/6 mice and Human embryonic kidney (HEK) 293 T cells purchased from the Cellular Institute in Shanghai, China. B16F10 cells were cultured at 37°C in 5% CO_2_ atmosphere in RPMI 1640 supplemented with 10% fetal bovine serum (FBS, Gibco BRL, USA) that contained 100U/ml penicillin G sodium and 100 μg/ml streptomycin. HEK 293 T cells were cultured in Dulbecco’s Modified Eagle Medium (DMEM, Invitrogen, NY, USA) plus 10% FBS, 2 mmol/L L-glutamine, 100 U/ml penicillin, and 100 μg/ml streptomycin at 37°C in a humidified 5% CO_2_ atmosphere.

### Short hairpin RNA sequence design, construction of shRNA1 targeting ZEB1 gene and screen of clones stably transfected with shRNA1

Short hairpin RNA sequence of mouse ZEB1 was designed based on the ZEB1 mRNA ID (GenBank NO.NM_011546.3) using the siDESIGN design software (Dharmacon, http://www.thermoscientificbio.com/design-center/) and Block-iTTM RNAi Designer (Invitrogen, Grand island, NY) as well as BLAST (http://www.ncbi.nlm.nih.gov/BLAST). ShRNA sequences are as follows: ZEB1-siRNA1: sense, 5′-*GATCC*CCATAGAGGCTACAA GCGCTTTA-*TTCAAGAGA*-TAAAGCGCTTGTAGCCTCTATTTTTTGGAAA-3′; antisense, 5′-*AGCTT*TTCCAAAAAATAGAGGCTACAAGCGCTTTA-*TCTCTTGAA*-TAAAGCGCTTGTAGCCTCTATGGG-3′. The square frames in nucleotide sequences contain the 9 nucleotide spacers, respectively. The underlined sequences contain *Bgl*II and *Hin*dIII enzyme cut sites. The primers were synthesized by Gene and Technology of China in Shanghai. A pSUPER-EGFP1 (enhanced green fluorescent protein 1) vector was used to construct recombinant pSUPER-EGFP1-ZEB1-shRNA1 as previously described [19]. A pSUPER- EGFP1-scrambled shRNA (Scramble) was used as a negative control. These recombinants were verified by the analyses of endonuclease digestion and sequencing. B16F10 cells were transfected with either the ZEB1-shRNAs or the Scramble-shRNA by using Lipofectamine™ 2000 reagent (Invitrogen, USA) following the manufacturer’s protocol. After an antibiotic selection with 800 μg/ml G418 (Clontech, CA), the ZEB1-shRNA1-B16F10 clones were isolated from the G418 resistant cells for each transfection pooled and expanded into cell lines and designated ‘B16F10/shZEB1’. ZEB1 expression was detected by quantitative-PCR and western blotting, respectively [20].

### Transduction of lentivirus miR-200c and production of stable expression clones

To generate the miR-200c expression lentivirus vector, we amplified an insert (full-length mouse miR-200c) by PCR from B16F10 cell DNA. The lentivirus miR-200c was produced from the transient transfection of the HEK293T cells with pHAGE-CMV-miR-200c-IZsGreen, psPAX2, and pMD2.G plasmid DNAs plus Lipofectamine 2000 (Invitrogen, USA) according to the manufacturer’s protocol. Forty-eight hours after the cotransfection, the lentivirus- bearing supernatants were collected and passed through a 0.45-mm filter. B16F10 cells were infected with the pHAGE-CMV-miR-200c-IzsGreen lentivirus, and were selected by the IzsGreen expression [21]. The stable expression clones were selected by limiting the dilution assay and designated‘B16F10/miR200c’ [22].

### Quantitative-PCR assay

Quantitative-PCR analysis was performed on an ABI step one plus real-time system (Applied Biosystems). The comparative Ct (ΔΔCt) method was used to determine the expression fold change [12]. Total cellular RNA was isolated from each sample by using a Qiagen RNeasy Kit (Qiagen, Valencia, CA). One microgram of total RNA from each sample was subjected to cDNA synthesis using the Superscript III reverse transcriptase (Invitrogen). cDNAs were amplified by PCR with primers as follows: miR-200c (sense, 5′-GAAGATCTGGAGCAGG AGATCTGCCGCTTC-3; reverse, GGAATTCAGAGCCACCCTTAACTCGG); ZEB1(sense, 5′-TGAGCACACAGGTAAGAGGCC-3′; reverse, 5′-GGCTTTTCCCCAGAGTGCA-3′); β-actin (sense, 5′-GCCCTGAGGCTCTTTTCCA −3′; reverse, 5′-TTACGGATGTCAACGTC A-3′); U6 (sense, 5′-CTCGCTTCGGCAGCACATAGG-3′; reverse, 5′-AACGCTTCACGAA TTTGCG TAGGAG-3′); URP Universal Reverse Primer, 5′-CCGGCAGGGTCCGAGGT-3′.

### Western blot

1 × 10^6^ different cells were collected and lyzed in the protein extraction buffer (Novagen, Madison, WI, USA) by following the manufacturer’s protocol. 12% sodium dodecyl sulfate-polyacrylamide gel electrophoresis was performed and the proteins (10 μg/lane) were transferred onto a PVDF membrane blocked with 4% dry milk in Tris-buffered saline with Tween-20 for 1 h at 20°C, and then incubated with the goat anti-mouse IL-21 (I-18, Santa Cruz Biotechnology Company, Santa Cruz, CA, USA), rabbit anti-mouse ZEB1, Vimentin, E-cadherin, N-cadherin, TGF-βand SMAD-7 (Bioworld Technology, Dublin, OH, USA), respectively for overnight at 4°C. The membrane was rinsed for 5 min with an antibody wash solution for 3 times before adding goat anti-rabbit or rabbit anti-goat fluorescence secondary antibody for 1 h at 20°C. Immunoreactive bands were detected by Odyssey scanning instrument (LI-COR Odyssey Imaging System, USA) [16].

### Animal experiment

C57BL/6 mice were initially immunized subcutaneous (s.c.) with 1 × 10^6^ B16F10/GPI-IL-21 tumor vaccine inactivated with 2 μg/ml mitoxantrone in a mouse’s right flank, and the immunization was performed three times at a week interval [16]. The B16F10/GPI-IL-21 tumor vaccine was developed as previously described [10] and stored in our Lab. About 10 days after the final immunization, the mice were randomly divided into four groups: the B16F10/wild type (WT) group; B16F10/shZEB1 group; B16F10/miR200 group and B16F10/GPI-IL-21 group. Finally, all mice were challenged s.c. with 2 × 10^5^ above-mentioned different B16F10 cells. Six mice/group were used in the study, and the experiment was repeated twice. Tumor growth was monitored every three day by measuring two perpendicular tumor diameters using calipers, and then the counts of lung metastases [15] were also examined after mice were sacrificed.

### Immunosorbent assay

Immunosorbent assay (ELISA) for detecting IFN-γ, TNF-α, IL-4 and TGF-β was performed by following the Kit’s protocol (eBioscience, CA). The Kit is suitable for detection of samples including cell culture supernatant and serum, and the sensitivity of Kit is 5.3 pg/mL [23,24].

### Assay of splenocyte cytotoxicity

Ten days after the final immunization, 5 × 10^6^ splenocytes were harvested from the mice immunized with the inactivated tumor vaccine B16F10/GPI-IL-21, and were used for effector cells that were labeled with 0.5 mM 5-(and 6)-carboxy-fluorescein diacetate succinimidyl ester (CFSE; 20 μg/ml) at 37°C for 15 min. The labeled splenocytes were washed twice in PBS containing 5% FBS to sequester any free CFSE. The CFSE-labeled effector cells were seeded with a constant number of B16F10 target cells in a 96-well plate at 25:1 ratios of effector cells to target cells (E:T). The cytotoxicity assay was performed in triplicate. Flow cytometric CFSE/7-AAD cytotoxicity assay was analyzed by Flow Cytometry (FCM, BD company, USA) [11,25].

### Immunohistochemistry

4 μm-thin formalin fixed and paraffin-embedded slides were incubated with the rabbit anti-mouse ZEB1, Vimentin, E-cadherin, N-cadherin, TGF-βand SMAD-7 (Bioworld Technology, Dublin, OH, USA), respectively overnight at 4°C. Antibody concentration was 1:1000. The samples were then labeled with horseradish peroxidase-conjugated streptavidin (Invitrogen) and the chromogenic reaction that was developed using Liquid DAB Substrate Pack according to the manufacturer’s instructions. The stained cells from random and non-overlapping fields were counted under a magnification of × 400 [15,26].

### Statistical analysis

Values of interest was presented as the mean plus or minus standard deviation. Statistical comparisons were performed using the Student’s *t*-test and Fisher/Chi-square test methods. A *P* value of <0.05 was considered statistically significant.

## Results

### Screen of transfected and transduced clones and identification of expression of IL-21, miR-200c and ZEB1 in a different B16F10 cells

**Figure 1:**
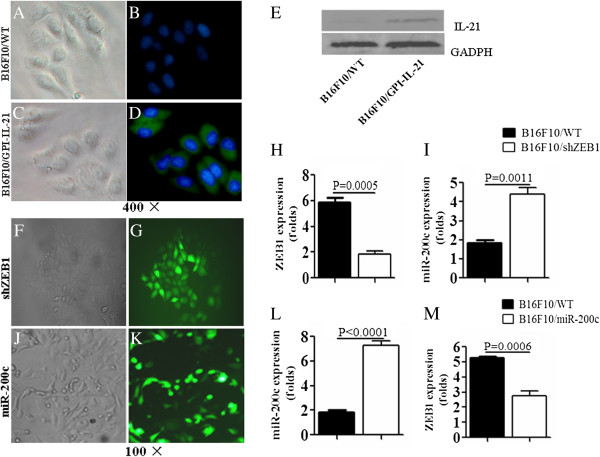
**Observation of the different clones and detection of expression of IL-21, miR-200c and ZEB1 in B16F10 cells. (A-D, F, G, J, K)** Morphological photos of B16F10 wild type (WT) clones, B16F10/GPI-IL-21 clones, B16F10/shZEB1 clones and B16F10/miR200c clones in order. The clones in left side were observed under a light microscrope, and the clones in right side were observed under a fluorescence microscrope. Magnification 400 × **(A-D)**, and magnification 100 × **(F, G, J, K). (D)** The fluorescence on the surface of B16F10/GPI-IL-21 cells, but no fluorescence on the surface of B16F10 WT cells **(B). (E)** The positive band IL-21 in lane 2 in B16F10/GPI-IL-21 cells detected by western blot. **(H, I)** The expression of ZEB1 RNA and miR200c in shZEB1 stably transfected B16F10/shZEB1 cells and B16F10 WT cells detected by qRT-PCR. **(L, M)** The expression of miR200c and ZEB1 in lentivirus miR-200c stably transduced B16F10/miR200c cells and B16F10 WT cells detected by qRT-PCR.

To develop B16F10/miR200c and B16F10/shZEB1, we first constructed the miR-200c lentivirus vector and shZEB1 recombinant and then transduced and transfected them into B16F10 cells, respectively, and finally the clones stably transduced with miR-200c lentivirus vector or transfected with shRNA1 recombinant were screened. Figures 1A-D show the B16F10 cells and the B16F10/IL-21-GPI cells were observed under a light microscope (left-panels) and under a fluorescence microscope(right-panels), in which the fluorescence is displayed on the surface of B16F10/IL-21-GPI cells, and the nucleus with DAPI (Figure 1D), but no green florescence on the surface of B16F10 cells in (Figure 1C). This result suggested IL-21 was expressed on the surface of B16F10/IL-21-GPI cells, which was further confirmed by the result of western blot. IL-21 band was found in B16F10/IL-21-GPI cells isolated from the G418 resistant cells as is shown in Figure 1E.

The B16F10/shZEB1 cell clones (Figure 1F) and the B16F10/miR200c cell clones (Figure 1J) were selected by limiting dilution assay, and were observed under a light microscope, and the same clones were observed under a fluorescence microscope (Figure 1G and K). The results suggested that the clones stably transfected with shRNA1 or stably infected with lentivirus miR-200c were successfully isolated from the B16F10 cells. After the endogenous expression of miR-200c or ZEB1 in the B16F10 cells was identified by RT-PCR (data not shown), we analyzed the expression of miR-200c and ZEB1 in the transfected and transduced B16F10 cells. It was found that the expression of ZEB1 was higher in the B16F10 wild type (WT) cells than in B16F10/shZEB1 cells, whereas the miR-200c expression was obviously increased in B16F10/ZEB1 cells compared with B16F10 WT cells (Figure 1H and I). Figure 1L indicates miR-200c expression is lower in the B16F10 WT cells than in B16F10/miR-200c cells, whereas the expression of ZEB1 was higher in the B16F10 WT cells than in B16F10/ miR-200c cells (Figure 1L and M). These molecular expression differences were statistically significant as are shown in Figure 1.

### Changes of serum cytokine levels and cytotoxicity of splenocytes in the mice immunized with the tumor vaccine B16F10/GPI-IL-21

**Figure 2:**
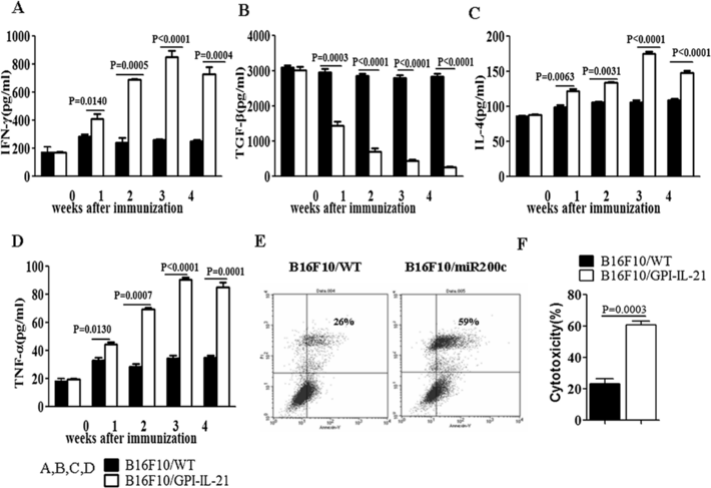
**Tumor vaccine B16F10/GPI-IL-21 induced immune responses in mice. (A-D)** Serum cytokine levels of IFN-γ, TNF-α, IL-4 and TGF-β were respectively detected by ELIAS in the B16F10/GPI-IL-21 vaccination of mice. **(E)** The splenocyte cytotoxicity to B16F10 cells at 25:1 ratio was analyzed by FCM in the immunized mice. **(F)** The analysis of splenocyte cytotoxicity.

To test the immune efficacy of the tumor vaccine B16F10/GPI-IL-21 we first detected serum cytokine levels in the immunized mice. Figures 2A-D indicate that compared with mice immunized with the B16F10 cells, the serum cytokine levels of IFN-γ, TNF-α and IL-4 were significantly increased after the mice were immunized with the tumor vaccine B16F10/GPI-IL-21, whereas the serum TGF-β level was markedly decreased, which was statistically significant (A-D). Next we analyzed the cellular immune response in the immunized mice. It was found that the cytotoxicity of splenocytes to B16F10 target cells at 25:1 ratio was notably enhanced (61±2.6%) in mice immunized with the tumor vaccine B16F10/GPI-IL-21 in contrast to mice immunized with the B16F10 cells (23±2.8%), and the difference was statistically significant (*P =* 0.0003). These data suggested that the tumor vaccine B16F10/GPI-IL-21 induced the mice to generate a strong immune responses.

### Tumor vaccine B16F10/GPI-IL-21 in combination with miR200c overexpression or ZEB1 knockdown reduced melanoma growth and metastasis

**Figure 3:**
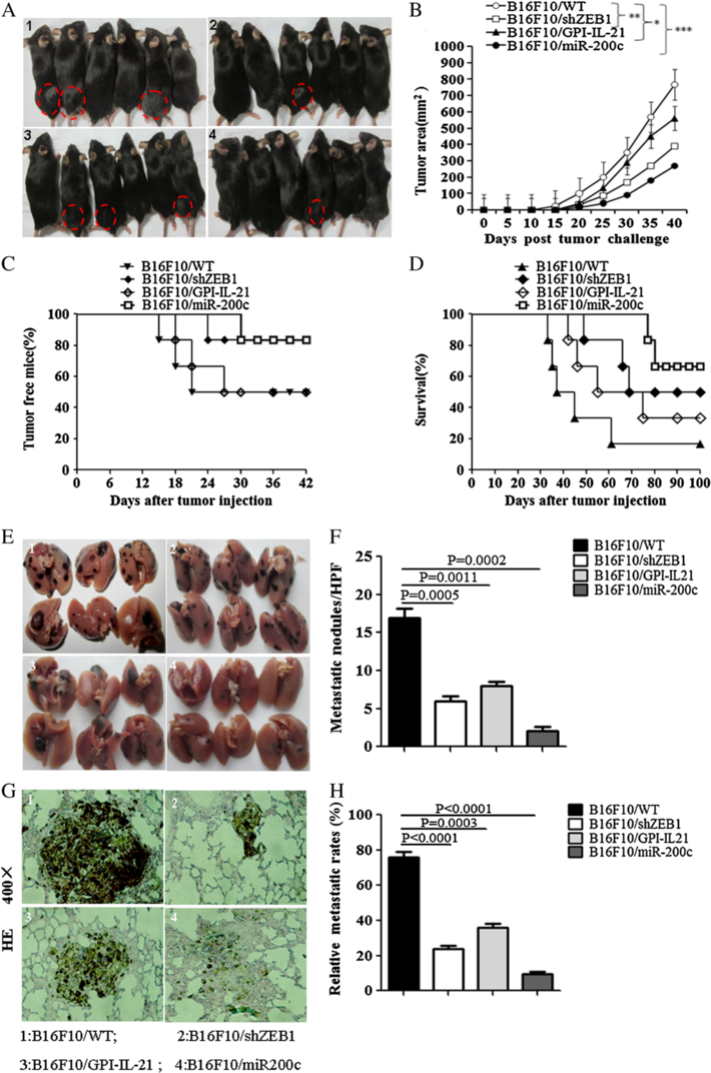
**B16F10/GPI-IL-21 vaccination of C57BL/6 mice in combination with either miR200c overexpression or ZEB1 knockdown against B16F10 melanoma. (A)** Images of the B16F10/ GPI-IL-21 vaccine immunized mice at 40 days after the mice were challenged by the different B16F10 cells. **(B-D)** Tumor volumes, tumor free mice and melanoma bearing mouse survival. **(E)** Images of metastasis nodules in murine lung. **(F)** The statistical analysis of metastatic tumor nodules. **(G)** The pathological changes in murine lung from the immunized mice that were challenged by the different B16F10 cells. **(H)** The statistical analysis of relative lung metastatic rates.

To augment therapeutic B16F10 melanoma efficacy by tumor vaccine B16F10/ GPI-IL-21 in C57BL/6 mice, we adopted a novel strategy that the tumor vaccine B16F10/GPI-IL-21 were combined with miR200c overexpression or ZEB1 knockdown in B16F10 cells in current antitumor experiment. Figure 3A gives the images of mice challenged by the different treated B16F10 cells at 40 days after the mice were immunized with the tumor vaccine B16F10/ GPI-IL-21 three times. We found that 3 of the 6 immunized mice developed tumors on Day 15, Day 18 and Day 21, respectively after the 6 mice were challenged by the B16F10 cells. The immunized mice challenged by the inactivated B16F10/GPI-IL-21tumor vaccine also developed tumors on Day 18, Day 21 and Day 27, respectively (3/6), but the tumor volumes were smaller than that of mice challenged by the B16F10 cells as is shown in Figure 3B.

Compared with the immunized mice that were challenged by the B16F10 cells, only 1 out of the 6 mice developed tumor after the mice were challenged by the B16F10/shZEB1 cells, and the measurable tumors were not detected in other 5 mice until 60 days into the observation, but the most powerful antimelanoma efficacy was found in the immunized mice that were challenged by the B16F10/miR200c cells, which was reflected in 1 out of the 6 mice developing the smaller tumor, the longer survival time, the lower tumor metastasis counts and the weaker pathological changes in murine lung than those of other mice that were shown in Figure 3. The results from the tumor area and tumor metastasis counts in lungs suggested that the synergism antitumor efficacy was found in mice immunized with the tumor vaccine B16F10/GPI-IL-21 in combination with either overexpression of miR200c or knockdown of ZEB1 in B16F10 cells.

### EMT-related molecular expression in tumor tissues in mice

**Figure 4:**
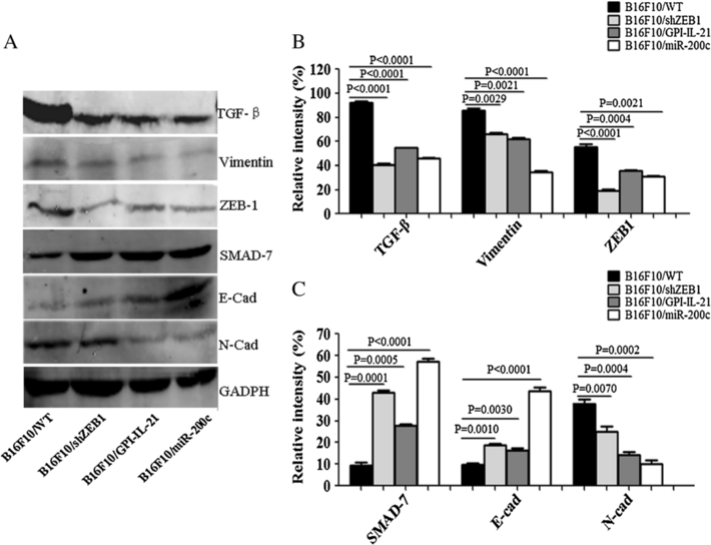
**EMT-related molecular expression detected by western blot. (A)** The EMT-related molecular expression was examined by western blot in B16F10 WT cells, B16F10/shZEB1 cells, B16F10/GPI-IL-21cells, and B16F10/miR200c cells, respectively. **(B, C)** The statistical analysis of relative intensity of molecular expression and the detection of the band intensities are all within the same dynamic range.

To analyze the mechanisms of the overexpression of miR200c or knockdown of ZEB1 for reinforcing the antimelanoma efficacy of the tumor vaccine B16F10/GPI-IL-21, we detected the EMT-related molecular expression in tumor tissues from the immunized mice. It is known that EMT is a process associated with many factors [12,17], of which TGF-β, Vimentin, ZEB-1, SMAD-7, E-cadherin and N-cadherin molecules are closely associated with the typical phenotype change of EMT in the process of tumor cell growth [22,27]. The representative results in Figure 4A showed these molecular expression in tumor tissues detected by western blotting.

**Figure 5:**
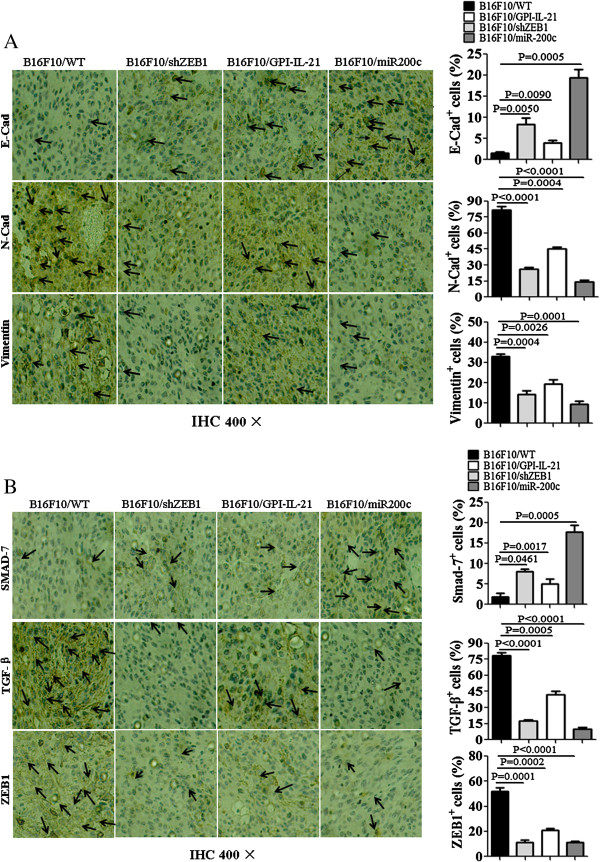
**EMT-related molecular changes detected by immunohistochemistry assay. (A, B)** The EMT-related molecular expression was examined by immunohistochemistry assay in B16F10 WT cells, B16F10/shZEB1 cells, B16F10/GPI-IL-21cells, and B16F10/miR200c cells, respectively. The statistical analysis of relative intensity of molecular expression were shown in histograms.

It was found that the expression of TGF-β, Vimentin, ZEB-1 and N-Cad was significantly decreased in tumor tissues from the B16F10/GPI-IL-21 vaccination of mice challenged by the B16F10/miR200c cells compared with other groups, whereas the expression of SMAD-7 and E-cadherin was significantly increased in tumor tissues, and the differences were statistically significant as are shown in Figures 4B-C. Consistent with the results of western blotting, the immunohistochemical analysis of tumor tissue sections showed that the molecular expression of TGF-β, Vimentin, ZEB-1 and N-cadherin was also reduced in tumor tissues, and that the SMAD-7 and E-cadherin expression was remarkably increased in the B16F10/GPI-IL-21 vaccination of mice that were then challenged by the B16F10/miR200c cells compared with B16F10/WT cells (Figure 5). From these results, we concluded that the B16F10/GPI-IL-21 vaccination of mice could result in the changes of EMT-related molecular expression in tumor tissues from the mice challenged by the B16F10/miR200c cells or the B16F10/shZEB1 cells that increased miR200c expression or decreased ZEB1 expression. The high expression of SMAD-7 and E-cadherin, accompanied with low expression of TGF-β, Vimentin, ZEB-1 and N-cadherin, may inhibit the EMT of B16F10 cells.

## Discussion

In the present study, our goal was to use a tumor vaccine B16F10/GPI-IL-21 in combination with regulation expression of miR200c and ZEB1 against melanoma, an aggressive skin cancer that there is no cure in advanced stages until now. We first selected the B16F10/GPI-IL-21 vaccine as an immune agonist to induce the mice to generate a strong immune responses such as increase of the serum cytokine levels of IFN-γ, TNF-α, IL-4, decrease of TGF-β level and enhancement of the splenocyte cytotoxic activity in the immunized mice. Since the malignant melanocytes can spread to distant locations and cause metastasis this process is involved in the EMT of melanoma in which miR200c and ZEB1 may be downregulation or upregulation. Therefore, we next combined the tumor vaccine B16F10/GPI-IL-21 with miR200c overexpression or ZEB1 knockdown in B16F10 cells to test the novel strategy for optimizing the therapeutic management of melanoma growth and metastasis in mice.

In an experimental model of melanoma growth and metastases, we found the tumor growth was significantly inhibited in the B16F10/GPI-IL-21 vaccination of mice that were then challenged by the B16F10/miR200c cells, which was reflected in weaker tumorigenicity, smaller tumor volumes, lower lung metastases and longer survival in melanoma bearing mice than those of mice that were challenged by the B16F10 cells (Figure 3). Even though, the B16F10/GPI-IL-21 vaccination of mice challenged by the B16F10/shZEB1 cells or B16F10/GPI-IL-21 cells also indicated antitumor efficacy this efficacy was remarkably found in B16F10/miR200c cell group.

To address the functional significance of the miR200c overexpression or ZEB1 knockdown in B16F10 cells, we tested the EMT-related molecular expression in tumor tissues. It is known that the increased expression of TGF-β, Vimentin, ZEB-1and N-cadherin could promote EMT progression of tumor cells [27-30], whereas the decreased expression of SMAD-7 and E-cadherin could inhibit the EMT progression [31,32]. It has been observed that the expression of TGF-β, Vimentin, ZEB-1 and N-cadherin detected by western blot was markedly decreased, accompanied with increased expression of SMAD-7 and E-cadherin in the B16F10/GPI-IL-21 vaccination of mice challenged by the B16F10/miR200c cells. Consistent with the western blot results, the expression of TGF-β, Vimentin, ZEB-1 and N-cadherin detected by immunohistochemistry assay was also remarkably decreased, whereas the expression of SMAD-7 and E-cadherin was notably increased in the tumor tissues. On the basis of these findings, we speculated that enforced miR-200c expression in melanoma B16F10 cells profoundly impairs cell tumorigenicity and phenotype change of EMT along with significantly decreased expression of TGF-β, Vimentin, ZEB-1and N-cadherin. Since the regulation of ZEB1 expression is also associated with the cell’s EMT, the tumor metastases and change of molecular expression above-mentioned were investigated in vivo experiment as well. We found that ZEB1 knockdown may correspond to reduced tumorigenicity and EMT change of B16F10 cells as well as melanoma metastases in vivo mouse model.

Several molecules, for example, IFN-gamma, SMAD-7 and E-cadherin confer inhibition of EMT to B16F10 cells [28,29,33,34]. It was reported that IFN-γ and TGF-β have opposite effects on diverse cellular functions. IFN-γ inhibits the TGF-β-induced phosphorylation of SMAD-3 and the accumulation of SMAD-3 in the nucleus, and the activation of TGF-β-responsive genes [33-35]. Based on the experiment results that the serum IFN-γ was increased, and TGF-β was decreased as well as SMAD-7 expression was increased in tumor tissues from the immunized mice, we presumed that IFN-γ may specifically inhibit an early step in the TGF-β-induced activation of SMAD-3 through a receptor serine kinase that phosphorylates and activates the transcription factors SMAD-2 and SMAD-3. Since IFN-γ also induces the expression of SMAD-7, an antagonistic SMAD, which prevents the interaction of SMAD-3 with the TGF-β receptor [33], therefore, we guess the increased SMAD-7 in tumor cells may bind to the TGF-β-receptor complex, preventing its interaction with, and phosphorylation of SMAD-3 [36,37], which may result in inhibition of melanoma growth and metastasis in mice. In addition, miR200c overexpression or ZEB1 knockdown is most likely to suppress tumor malignancy by enhancing E-cadherin expression (Figures 4 and 5A) and/or by inhibiting signals that suppress E-cadherin function.

## Conclusions

Our data represents the first attempt to improve the tumor vaccine B16F10/GPI-IL-21 efficacy in combination with miR200c overexpression or ZEB1 knockdown in B16F10 cells. This efficacy resulted in inhibition of melanoma growth and metastasis in melanoma bearing mouse model. The tumor vaccine B16F10/GPI-IL-21 improves IFN-γ secretion and induces antitumor immune responses. Regulation expression of miR200c and ZEB1 in B16F10 cells positively led to expression changes of TGF-β, Vimentin, ZEB-1, N-cadherin, SMAD-7 and E-cadherin in tumor tissues from the B16F10/GPI-IL-21 vaccination of mice, which are correlated with progression of EMT of B16F10 cells as well as influence of melanoma growth and metastasis in mice. These findings encourage the exploitation of these combined strategies in clinic trials.

## Abbreviations

EMT: Epithelial to mesenchymal transition; GPI: Glycosylpho sphatidylinositol; miR200c: microRNA-200c; ZEB1: Zinc-finger E-box binding homeobox 1; CFSE: Carboxyfluoroscein diacetate succinimidyl ester; FACS: Fluorescence-assisted cell; ELISPOT: Enzyme-linked immunospot; TGF-β: Transforming growth factor-β; IFN-γ: Interferonγ; TNF-α: Tumor necrosis factor α; IL-4: Interleukin 4.

## Competing interests

The authors declared that they have no competing interests.

## Authors’ contributions

XW, FZ and XH carried out the experiments described in the manuscripts, developed the technique described in the manuscript, and participated in the writing of the manuscript. JW, HZ, FS, YZ, KC and JS participated in most of the experiments. JD and FZ contributed to the design of the experiments and contributed to the writing of the manuscript. All authors have read and approved the final manuscript.

## Authors’ information

Xiaoying Wang, Xiangfeng He and Fengshu Zhao are co-authors.
